# Defining a Healthy Diet: Evidence for the Role of Contemporary Dietary Patterns in Health and Disease

**DOI:** 10.3390/nu12020334

**Published:** 2020-01-27

**Authors:** Hellas Cena, Philip C. Calder

**Affiliations:** 1 Laboratory of Dietetics and Clinical Nutrition, Department of Public Health, Experimental and Forensic Medicine, University of Pavia, 27100 Pavia, Italy; 2 Clinical Nutrition and Dietetics Service, Unit of Internal Medicine and Endocrinology, ICS Maugeri IRCCS, 27100 Pavia, Italy; 3 Human Development and Health, Faculty of Medicine, University of Southampton, Southampton SO16 6YD, UK; 4 NIHR Southampton Biomedical Research Centre, University Hospital Southampton NHS Foundation Trust and University of Southampton, Southampton SO16 6YD, UK

**Keywords:** healthy dietary patterns, non-communicable diseases, macronutrients, micronutrients, non-essential nutrients, plant-based diets

## Abstract

The definition of what constitutes a healthy diet is continually shifting to reflect the evolving understanding of the roles that different foods, essential nutrients, and other food components play in health and disease. A large and growing body of evidence supports that intake of certain types of nutrients, specific food groups, or overarching dietary patterns positively influences health and promotes the prevention of common non-communicable diseases (NCDs). Greater consumption of health-promoting foods and limited intake of unhealthier options are intrinsic to the eating habits of certain regional diets such as the Mediterranean diet or have been constructed as part of dietary patterns designed to reduce disease risk, such as the Dietary Approaches to Stop Hypertension (DASH) or Mediterranean-DASH Intervention for Neurodegenerative Delay (MIND) diets. In comparison with a more traditional Western diet, these healthier alternatives are higher in plant-based foods, including fresh fruits and vegetables, whole grains, legumes, seeds, and nuts and lower in animal-based foods, particularly fatty and processed meats. To better understand the current concept of a “healthy diet,” this review describes the features and supporting clinical and epidemiologic data for diets that have been shown to prevent disease and/or positively influence health. In total, evidence from epidemiological studies and clinical trials indicates that these types of dietary patterns reduce risks of NCDs including cardiovascular disease and cancer.

## 1. Introduction

Non-communicable diseases (NCDs) such as cardiovascular disease, cancer, chronic respiratory diseases, diabetes, obesity, and cognitive impairment are among the leading causes of death and disability throughout the world, affecting populations in developed as well as developing countries [1]. Although there are established genetic and environmental contributors to NCD risk, modifiable lifestyle-related factors play a large role at the individual level [2,3,4]. Dietary choices, for example, contribute to the risk for developing hypertension, hypercholesterolemia, overweight/obesity, and inflammation, which in turn increase the risk for diseases that are associated with significant morbidity and mortality, including cardiovascular disease, diabetes, and cancer [5]. Indeed, the marked rise in chronic NCDs has a causal link to global dietary patterns that are becoming increasingly Westernized [6], being characterized by high levels of fatty and processed meats, saturated fats, refined grains, salt, and sugars but lacking in fresh fruits and vegetables.

In recognition of the importance of the diet as a determinant of disease risk, the World Health Organization (WHO) Global Action Plan for the Prevention and Control of Noncommunicable Diseases includes strategies for addressing unhealthy diet patterns among its initiatives directed at reducing behavioral risk factors; the other components comprise physical inactivity, tobacco use, and harmful alcohol use [1]. Dietary changes recommended by WHO include balancing energy intake, limiting saturated and trans fats and shifting toward consumption of unsaturated fats, increasing intake of fruits and vegetables, and limiting the intake of sugar and salt. Many of these dietary targets naturally occur in regional diets such as the Mediterranean diet [7] or are included as part of evidence-based diets designed to reduce disease risk, such as the Dietary Approaches to Stop Hypertension (DASH) [8] or Mediterranean-DASH Intervention for Neurodegenerative Delay (MIND) [9] diets. To better understand the current concept of a “healthy diet”, this narrative review describes the features and supporting clinical and epidemiologic data for diets that align with the general WHO guidance and have been shown to prevent disease and/or positively influence health.

## 2. Components of a Healthy Diet and Their Benefits

A healthy diet is one in which macronutrients are consumed in appropriate proportions to support energetic and physiologic needs without excess intake while also providing sufficient micronutrients and hydration to meet the physiologic needs of the body [10]. Macronutrients (i.e., carbohydrates, proteins, and fats) provide the energy necessary for the cellular processes required for daily functioning [11]. Micronutrients (i.e., vitamins and minerals) are required in comparatively small amounts for normal growth, development, metabolism, and physiologic functioning [12,13].

Carbohydrates are the primary source of energy in the diet and are found in the greatest abundance in grains, fruits, legumes, and vegetables [14]. In terms of deriving a health benefit, whole grains are preferred over processed grains, the latter having been stripped of germ and bran during the milling process, resulting in lower amounts of fiber and micronutrients [15]. Meta-analyses of prospective cohort studies have linked increased whole-grain intake to a reduced risk of coronary heart disease, stroke, cardiovascular disease, and cancer, as well as to the decreased risk of mortality due to any cause, cardiovascular disease, cancer, respiratory disease, diabetes, and infectious disease [15,16,17]. Fresh fruits and vegetables supply energy as well as dietary fiber, which promotes the feeling of satiety and has positive effects on gastrointestinal function, cholesterol levels, and glycemic control [18]. In addition, fresh fruits and vegetables are key sources of phytochemicals (e.g., polyphenols, phytosterols, carotenoids), which are bioactive compounds believed to confer many of the health benefits associated with fruit and vegetable consumption [19]. The mechanistic effects of these various phytochemicals are unclear but include their antioxidative properties, as well as their role in regulating nuclear transcription factors, fat metabolism, and inflammatory mediators. For example, flavonoids have been shown to increase insulin secretion and reduce insulin resistance, suggesting that these phytochemicals provide some benefits in obesity and diabetes [20]. Additionally, polyphenols interact with gastrointestinal microbiota in a bi-directional manner by enhancing gut bacteria and being metabolized by these bacteria to form more bioactive compounds [20]. Fruit and vegetable intake has been shown to inversely correlate with the risk of NCDs, including hypertension [21], cardiovascular disease [22,23], chronic obstructive pulmonary disease [24], lung cancer [25], and metabolic syndrome [26].

Dietary proteins provide a source of energy as well as amino acids, including those that the human body requires but cannot produce on its own (i.e., essential amino acids). Dietary proteins are derived from both animal (meat, dairy, fish, and eggs) and plant (legumes, soya products, grains, nuts, and seeds) sources, with the former considered a richer source due to the array of amino acids, high digestibility, and greater bioavailability [27]. However, animal-based sources of protein contain saturated fatty acids, which have been linked to cardiovascular disease, dyslipidemia, and certain cancers. Although the mechanisms are unclear, red meat, and processed meat in particular, have been associated with an increased risk of colorectal cancer [28,29]. Animal-derived proteins also increase the dietary acid load, tipping the body’s acid-base balance toward acidosis [30,31]. The increased metabolic acid load has been linked to insulin resistance, impaired glucose homeostasis, and the development of urinary calcium stones [30,31].

Adequate dietary protein intake is important for maintaining lean body mass throughout the life span. In older adults, protein plays an important role in preventing age-related loss of skeletal muscle mass [32], preserving bone mass, and reducing fracture risk [33]. For older individuals not obtaining adequate protein from their diets, supplementation with amino acids can improve strength and functional status [34].

Fats (or lipids) are the primary structural components of cellular membranes and are also sources of cellular energy [35]. Dietary fats fall into 4 categories: monounsaturated fats, polyunsaturated fats, saturated fats, and trans fats. The fat content of food is generally an admixture of these different types [35]. Unsaturated fats are found in a variety of foods, including fish, many plant-derived oils, nuts, and seeds, whereas animal products (and some plant-derived oils) contribute a larger proportion of saturated fats [35,36]. Trans fats found in foods are predominantly the result of processing vegetable oils but are also present in small quantities in animal products (i.e., ruminant trans fats from cows, sheep, and goats) [35,36]. Among the types of dietary fats, unsaturated fats are associated with reduced cardiovascular and mortality risks, whereas trans fats and, to a lesser degree, saturated fats are associated with negative impacts on health, including increased mortality risk [36,37]. Two families of polyunsaturated fatty acids, omega-3 and omega-6, are described as essential fatty acids, because they are required for normal growth and reproduction but are not produced by the body and, therefore, must be obtained from dietary sources [10]. Omega-3 fatty acids, in particular, eicosapentaenoic acid (EPA), and docosahexaenoic acid (DHA), have been widely studied for their potential health benefits, with evidence suggesting positive effects including cardioprotection, preventing cognitive decline, reducing inflammation, sustaining muscle mass, and improving systemic insulin resistance [38,39,40]. Seafood, especially oily fish, provides EPA and DHA, and supplements are widely available for those not meeting recommended intakes with diet alone [41,42]. Nuts and some seeds and plant oils provide alpha-linolenic acid, the major plant omega-3 fatty acid [43].

Although required in trace amounts compared with macronutrients, micronutrients are necessary for normal growth, metabolism, physiologic functioning, and cellular integrity [12,13]. The shift from whole foods to processed, refined foods has reduced the micronutrient quality of the modern Western diet [44]. Vitamin and mineral inadequacies have been implicated in cellular aging and late-onset disease, as scarcity drives chronic metabolic disruption. Keeping with these observations, adequate dietary intake of, or supplementation with, micronutrients that have antioxidant properties (e.g., vitamins A, C, and E, copper, zinc, and selenium) has been suggested as a means to reduce the risk for and progression of age-related diseases [45].

Water is the principal component of the body, constituting the majority of lean body mass and total body weight [13]. Water not only provides hydration but also carries micronutrients, including trace elements and electrolytes [46,47]. Drinking water may supply as much as 20% of the daily recommended intake of calcium and magnesium [47]. Our understanding of water requirements and water’s effect on health and disease is limited, although the global increase in intake of high-calorie beverages has refocused attention on the importance of water for maintaining health and preventing disease [46].

## 3. Common Health-Promoting Dietary Patterns 

**Figure 1:**
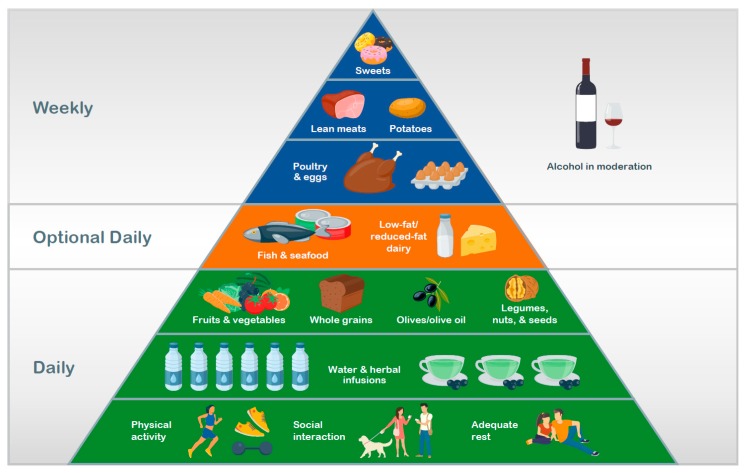
A generalized healthy diet and lifestyle pyramid.

Based on our understanding of nutritional requirements and their likely health impacts as described above, healthy dietary patterns can be generally described as those that are rich in health-promoting foods, including plant-based foods, fresh fruits and vegetables, antioxidants, soya, nuts, and sources of omega-3 fatty acids, and low in saturated fats and trans fats, animal-derived proteins, and added/refined sugars [48]. Patterns such as these are naturally occurring in certain regions of the world and rooted in local/regional tradition and food sources, as is the case for the traditional Mediterranean and Asian diets. Healthy dietary patterns have also been developed based on studies of nutrient intake and subsequent health measures or outcomes (e.g., the DASH [8] and MIND [9] diets) that share some common characteristics (Figure 1).

### 3.1. Mediterranean Diet

The Mediterranean diet is based on components of the traditional dietary patterns of Euro-Mediterranean countries and encompasses not only the types of foods consumed and their relative contributions to daily nutrient intake, but also an approach to eating that is cognizant of how foods are sourced (e.g., sustainability and eco-friendliness), cooked, and eaten, as well as lifestyle considerations such as engaging in regular physical activity, getting adequate rest, and participating in fellowship when preparing and sharing meals [7]. Within the core framework of the Mediterranean diet, variations based on geography and culture are reflected in the emphasis on the inclusion of traditional and local food products. The primary basis of daily meals in the Mediterranean diet is cereals such as whole-grain bread, pastas, couscous, and other unrefined grains that are rich in fiber and a variety of fruits and vegetables of different colors and textures that are high in micronutrients, fiber, and phytochemicals (Table 1) [7,9,49,50,51,52]. Dairy products, preferably low-fat yogurt, cheese, or other fermented dairy products, are recommended daily in moderation as a source of calcium, which is needed for bone and heart health. Olive oil serves as the primary source of dietary lipids and is supplemented with olives, nuts, and seeds. Water (1.5–2.0 L/day or ~8 glasses) is recommended as the main source of hydration, whereas wine and other fermented alcoholic beverages are generally permitted in moderation, to be consumed with meals. Fish, white meat, and eggs are the primary sources of protein; red meat and processed meats are consumed less frequently and in smaller portions. Legumes are also a preferred source of plant-based proteins [7].

The health benefits of the Mediterranean diet were first described in 1975 by Ancel Keys, who observed a reduction in cardiovascular disease risk among populations whose nutritional model was consistent with practices of peoples from the Mediterranean Basin [53]. Since that time, research has revealed beneficial effects of the Mediterranean diet on a number of NCDs and related health measures, including cardiovascular and cerebrovascular disease [54], cancer [55], glycemic control [56], and cognitive function [57,58]. Although publication of a key intervention study (Prevención con Dieta Mediterránea; PREDIMED) conducted at multiple sites across Spain and evaluating the Mediterranean diet for the primary prevention of cardiovascular disease was retracted due to irregularities in randomization [59], a subsequent analysis adjusting for these issues reported a consistent positive effect of adhering to a Mediterranean diet supplemented with olive oil or nuts compared with a reduced-fat diet [59]. Substudies of PREDIMED have also shown that, compared with a low-fat control diet, the Mediterranean diet supplemented with olive oil or nuts is associated with a 30% reduced risk of major cardiovascular risk events [59] and reductions in systolic blood pressure (SBP) and diastolic blood pressure (DBP) of 5.8–7.3 mmHg and 3.3–3.4 mmHg, respectively [60]. In addition, cardiovascular factors such as mean internal carotid artery intima-media thickness (−0.084 mm; *p* < 0.05) and maximum plaque height (−0.091 mm; *p* < 0.05) are improved with the Mediterranean diet supplemented with nuts [61]. Greater intake of polyphenols (phytochemicals found in fruits, vegetables, tea, olive oil, and wine) correlated with a 36% reduced risk of hypertension (*p* = 0.015) [62] and improvements in inflammatory biomarkers related to atherosclerosis (i.e., interleukin [IL]-6, tumor necrosis factor-alpha, soluble intercellular adhesion molecule-1, vascular cell adhesion molecule-1, and monocyte chemotactic protein-1; *p* < 0.05 for each), as well as in high-density lipoprotein cholesterol (HDL-C; *p* = 0.004) [62,63].

### 3.2. Dietary Approaches to Stop Hypertension (DASH)

The DASH diet derives its name from the Dietary Approaches to Stop Hypertension study, which evaluated the influence of dietary patterns on blood pressure [8]. Patients who consumed a diet that was rich in fruits, vegetables, and low-fat dairy and that included a reduced amount of saturated and total fat and cholesterol experienced significantly greater reductions in blood pressure than patients who consumed a control diet that was similar in composition to a typical American diet (difference in SBP/DBP, −5.5/−3.0 mmHg; *p* < 0.001) or a diet rich in fruits and vegetables with a reduced amount of snacks and sweets (−2.7/−1.9 mmHg; *p* ≤ 0.002). All 3 diets had a sodium content of 3 g per day. A subsequent study (DASH-Sodium) that explored the DASH diet or a control diet in combination with varying levels of sodium intake (high, intermediate, and low) found that the DASH diet significantly reduced SBP during the high, intermediate, and low sodium intake phases of both diets (high: −5.9 mmHg; *p* < 0.001; intermediate: −5.0 mmHg; *p* < 0.001; low: −2.2 mmHg; *p* < 0.05) [64]. The DASH diet also significantly reduced DBP versus the control diet during the high (−2.9 mmHg; *p* < 0.001) and intermediate (−2.5 mmHg; *p* < 0.01) sodium intake phases but not during the low intake phase (−1.0 mmHg). Although reducing sodium intake also significantly reduced blood pressure in the control diet group (*p* < 0.05), the low sodium phase of the DASH diet elicited significant decreases in SBP/DBP of −8.9/−4.5 mmHg (*p* < 0.001 for each) compared with high sodium intake phase of the control diet.

Subsequent controlled trials, as a whole, support the results of the DASH and DASH-Sodium studies in terms of blood pressure reduction. Moreover, these studies expanded the positive impacts of the DASH diet to include improvements in other cardiovascular risk factors or comorbidities (e.g., low-density lipoprotein cholesterol [LDL-C], total cholesterol, overweight/obesity, and insulin sensitivity) [65,66,67,68] and reductions in adverse outcomes such as development of cardiovascular disease, coronary heart disease, stroke, heart failure, metabolic syndrome, and diabetes (including improved pregnancy outcomes in women with gestational diabetes) [68,69,70,71,72]. Meta-analyses of studies using the DASH diet have demonstrated that LDL-C is significantly reduced by −0.1 mmol/L (*p* = 0.03) [65,68], total cholesterol by −0.2 mmol/L (*p* < 0.001) [65,68], body weight by −1.42 kg (*p* < 0.001) [66,68], and fasting insulin by −0.15 μU/mL (*p* < 0.001) [65,66,67,68]. With the DASH diet, the risk of cardiovascular disease is reduced by 20%, stroke by 19%, and heart failure by 29% (*p* < 0.001 for each) [69,71]. The overall risk of diabetes is reduced by 18% [68], and children and adolescents with higher DASH scores (i.e., those whose diets included the highest intakes of fruits, vegetables, nuts, legumes, low-fat dairy, and whole grains) were at 64% lower risk of developing metabolic syndrome than those with the lowest DASH scores (*p* = 0.023) [71]. Furthermore, rates of cesarean section decreased by 47% [72], incidence of macrosomia (birth weight > 4000 g) decreased from 39% to 4% (*p* = 0.002) [70], and significantly fewer women experienced gestational diabetes that required insulin therapy on the DASH diet (23%) compared with the control diet (73%; *p* < 0.0001) [70].

The dietary pattern derived from the DASH study emphasizes the consumption of an array of vegetables (including colorful varieties, legumes, and starchy vegetables), fruits, fat-free or low-fat dairy products, whole grains, and various protein sources (e.g., seafood, lean meats, eggs, legumes, nuts, seeds, and soya) (Table 1) [49]. Limited consumption of added sugars (< 10% of calories per day), saturated fats (< 10% of calories per day), sodium (< 2300 mg/day), and alcohol (≤ 1 drink per day for women and ≤ 2 drinks per day for men) is suggested. In addition, further reductions in blood pressure may be achievable by further reducing sodium intake, although practical challenges may limit the ability to achieve sodium intake of 1200 mg or less per day [49].

### 3.3. Mediterranean-DASH Intervention for Neurodegenerative Delay (MIND)

The MIND diet combines elements of the Mediterranean and DASH diets with the goal of sustaining cognitive health throughout older age [9]. Both the Mediterranean and DASH diets have been individually linked to positive cognitive outcomes, including the prevention of cognitive decline or impairment and better cognitive performance [73,74,75]. Two high-quality cohort studies have reported associations between adherence to the MIND diet and a 53% lower risk for developing Alzheimer’s disease (*p* = 0.002 for linear trend) [50] and slower declines in cognitive functioning, both overall and within specific cognitive domains (e.g., episodic, semantic, and working memory and perceptual speed and organization), such that the highest adherence rates to the MIND diet were associated with cognitive function equivalent to being 7.5 years younger [50,76]. Interestingly, even modest adherence to the MIND diet was associated with a 35% risk reduction for Alzheimer’s disease versus the lowest adherence group (*p* = 0.002 for linear trend), whereas high adherence was needed to demonstrate 54% and 39% risk reductions with the Mediterranean and DASH diets, respectively; high adherence to the Mediterranean and DASH diet showed a statistically significant benefit [50].

The MIND diet focuses on increasing the intake of fresh fruits and vegetables and emphasizes brain-healthy foods such as green leafy vegetables, nuts, berries, beans, whole grains, fish, poultry, olive oil, and wine in moderation (Table 1) [9,50]. Additionally, foods that are thought to be unhealthy for the brain, such as red meats, butter/margarine, cheese, pastries, sweets, and fried or fast food, are limited [9]. The specificity regarding the types of foods on the healthy and unhealthy lists differentiates MIND from the Mediterranean or DASH diets [50].

### 3.4. Nordic Diet

Iterations of a Nordic diet (e.g., the healthy Nordic diet, New Nordic Diet) arose from the desire to translate the Mediterranean, DASH, and other health-promoting diets into a regionally tailored dietary pattern that uses traditional, local Nordic foods and would be attractive to the public, sustainable, and eco-friendly [77,78]. Overarching tenets of the New Nordic Diet are to consume more (1) calories from plant sources and fewer from animal sources, (2) foods from seas and lakes, and (3) foods from the wild countryside [78,79]. A generalized Nordic dietary pattern would include green leafy vegetables, other vegetables, fruits, fish and seafood, potatoes, berries, whole grains (e.g., wheat, rye, oats, barley), nuts, low-fat dairy products, rapeseed, sunflower, and/or soya oils and limited intake of fresh red meat and sugar [78,80]. Specific dietary recommendations based on the NORDIET clinical trial are presented in Table 1 [51].

The randomized, controlled NORDIET study compared a healthy Nordic diet with a control diet (the participant’s usual Western diet) [77]. Over 6 weeks, the Nordic diet improved the lipid profile (including a 0.98 mmol/L reduction in total cholesterol [*p* < 0.0001] and a 0.83 mmol/L reduction in LDL-C [*p* < 0.001]), lowered SBP by 6.6 mmHg (*p* = 0.008), and improved insulin sensitivity (homeostatic model assessment-insulin resistance decreased 0.11; *p* = 0.01) compared with the control diet. Those on the Nordic diet also experienced a 3.0 kg decrease in body weight (*p* < 0.001) despite food being available ad libitum.

Results from subsequent studies conducted using Nordic diet variations are consistent with those from studies with the NORDIET study, demonstrating improvements relative to the control diet in blood lipid profile (LDL-C/HDL-C ratio, −0.15; *p* = 0.046) [81], inflammation (IL-1 receptor antagonist, −84 ng/L; *p* < 0.001) [81], blood pressure (DBP, −4.4 mmHg (*p* = 0.001), and mean arterial pressure (−4.2 mmHg; *p* = 0.006) among patients with metabolic syndrome [82] and weight loss (−3.22 kg; *p* < 0.001) [83] and blood pressure reduction (SBP/DBP, −5.13/−3.24 mmHg; *p* < 0.05) in individuals with obesity [83]. Compared with baseline values, one study demonstrated blood pressure reductions of −6.9 mmHg (SBP) and −3.2 mmHg (DBP; *p*< 0.01) [83,84]. Additionally, a study conducted in children reported an improvement in omega-3 fatty acid status with the Nordic diet that was associated with improvements in school performance (*p* < 0.05) [85]. A systematic review parsing the individual components of the Nordic diet found that evidence supported the protective effects of eating whole grains on type 2 diabetes and cardiovascular disease risk, but that there was insufficient evidence for other foods in the Nordic diet [86].

### 3.5. Traditional Asian Diets 

Although there is substantial evidence supporting the Mediterranean and other European-based diets, traditional regional dietary patterns from other parts of the world that follow similar principles have less–well-established links to positive health outcomes. A full description of the breadth of regional diets and the associated evidence bases is beyond the scope of this publication, but we consider some Asian-based diets to be particularly relevant to this discussion.

The traditional Korean diet is composed of rice and other whole grains, fermented food, indigenous land and sea vegetables, proteins primarily from legumes and fish as opposed to red meat, medicinal herbs (e.g., garlic, green onions, ginger), and sesame and perilla oils [87]. Meals typically consist of multiple small-portion dishes are often derived from seasonal food sources and are home-cooked. Unlike the Western diet, the traditional Korean diet does not include many fried foods [87]. Epidemiologic data suggest a reduced risk of metabolic syndrome (odds ratio [OR]: 0.77; 95% CI: 0.60–0.99), obesity (OR: 0.72; 95% CI: 0.55–0.95), hypertension (OR: 0.74; 95% CI: 0.57–0.98), and hypertriglyceridemia (OR: 0.76; 95% CI: 0.59–0.99) among individuals who follow traditional Korean dietary patterns [88]. These findings are consistent with a controlled clinical trial that explored the effects of a traditional Korean diet compared with a control diet (“eat as usual”) on cardiovascular risk factors in patients with diabetes and hypertension. In that study, adherence to a traditional Korean diet favorably influenced body composition (body weight, −2.3 kg; body mass index [BMI], −0.83 kg/m^2^; body fat, −2.2%; *p* < 0.01), heart rate (−7.1 bpm; *p* = 0.002), and glycemic control (HbA1c, −0.72%; *p* = 0.003) [89].

The traditional Chinese diet features rice or noodles, soups, vegetables, steamed breads or dumplings, fruits and vegetables, soy, seafood, and meat [90,91]. Although higher in carbohydrates and lower in fat compared with a Western diet, the traditional Chinese diet does not appear to promote weight gain in healthy, normal-weight Chinese, suggesting that carbohydrate restriction may not be a universally applicable intervention to combat obesity and cardiometabolic risk [92]. One 6-week controlled trial demonstrated that 52% of non-Chinese individuals with overweight or obesity who adhered to a traditional Chinese diet had a reduction in BMI while preserving lean body mass compared with 28% of those who followed a Western diet at the 1-year follow-up assessment [93]. In another trial, BMI decreased by 0.37 kg/m^2^ and lean mass by 0.21 kg among subjects who adhered to a traditional Chinese diet for 6 weeks, whereas those who followed a Western diet had 0.26 kg/m^2^ and 0.49 kg reductions in BMI and lean body mass, respectively [94]. Notably, both of these studies restricted caloric intake to 1,200 Kcal for the test and control diet groups.

Similar to the Korean diet, the traditional Japanese diet (known as Washoku) is characterized by small portions of multiple components, primarily including rice, fish (often eaten raw), soups, and pickles [95]. Fermented soybean paste (dashi) serves as the base of many of the soups that are central to the traditional Japanese diet; other ingredients include seaweed, fruits and vegetables, and mushrooms. The use of chopsticks, alternating between dishes of small portion size throughout a meal, and the base flavor of Japanese food (umami) enhance satiety and help to prevent overeating. Adherence to a traditional Japanese dietary pattern has been associated with favorable effects on blood pressure among apparently healthy Japanese adults [96]. This is consistent with data from the 2012 Japan National Health and Nutrition Survey demonstrating that adherence to a traditional Japanese diet compared with a Western diet or a meat- and fat-based dietary pattern was associated with a lower prevalence of hypertension in men [97]. However, in the same study, a traditional Japanese diet was associated with higher DBP in women, as well as higher waist circumference and BMI in men. Further study is needed to elucidate the health impacts of traditional Japanese and other Asian dietary patterns.

## 4. Additional Factors 

While the evidence reviewed here suggests that the described dietary patterns positively influence measures of health and disease risk and outcome because they encourage the intake of foods that individually have beneficial effects and the avoidance of unhealthy options, additional factors combine to create a lifestyle that promotes health. For example, healthy diets include adequate hydration, typically in the form of water or tea/herbal infusions [7,49,51,52]. In addition to the dietary components, a healthy lifestyle is one that incorporates regular exercise, socialization, and adequate sleep [7,52], and minimizes elements that have a negative effect on health such as tobacco use, excessive alcohol consumption, physical inactivity, large amounts of screen time, and stress.

The importance of non-dietary factors is reflected in their inclusion in modern food pyramids. Built on a base of positive lifestyle factors, the lower tiers indicate daily consumption of adequate hydration and nutrient-rich, plant-based foods, with animal-derived products (meat, fish, and dairy) and sweets comprising higher tiers of the pyramid (i.e., less frequently or infrequently consumed items).

Whereas the goal may be to achieve nutrient requirements through food and water intake alone, there are situations in which food-derived nutrient intake might be inadequate due to increased need, selective eating, or food insecurity/limited access to more nutritious foods [98,99,100]. Therefore, for some individuals, dietary supplements may be required, particularly at certain life phases. For example, later in life, the recommended intake of calcium increases to sustain bone mineral density [101]; hence, supplementation with calcium may be necessary to meet recommended intake levels in older adults. Before initiating supplementation, dietary intake levels should be considered to avoid exceeding the upper tolerability limits and causing adverse events.

There are a number of other traditional regional diets that likely have similar benefits to those that we describe here. However, we made the decision to narrow our focus to those diets with evidence from randomized, controlled trials demonstrating their health benefits. For example, the African Heritage Diet focuses on traditional ingredients that may be beneficial to African American populations who experience disproportionately higher risks for chronic diseases related to their diets [102]. Future research is warranted to evaluate the impact of the African Heritage Diet and other regional dietary patterns on health.

## 5. Conclusions

Healthy diets, arising either by tradition or design, share many common features and generally align with the WHO Global Action Plan for the Prevention and Control of Noncommunicable Diseases. In comparison with a Western diet, these healthier alternatives are higher in plant-based foods, including fresh fruits and vegetables, whole grains, legumes, seeds, and nuts and lower in animal-based foods, particularly fatty and processed meats. Evidence from epidemiologic studies and clinical trials indicates that these types of dietary patterns reduce risks of NCDs ranging from cardiovascular disease to cancer. Further endeavors are needed to integrate these healthy dietary and lifestyle choices into daily living in communities throughout the world and to make healthy eating accessible, achievable, and sustainable.

## Figures and Tables

**Table 1:** Comparison of nutritional/lifestyle components among different healthy diet options.

Dietary Component	Recommended Servings				
	Mediterranean [7]	DASH [49] ^a^	MIND [9,50]	Healthy Nordic [51]	Traditional Asian [52]
**Fruits**	1–2/meal	4–5 servings/day	Berries: ≥ 2 servings/week	Fruits, berries, vegetables, and potatoes: ≥ 500 g/day	Daily
**Vegetables**	≥ 2 servings/meal	4–5 servings/day	Green leafy: ≥ 6 servings/week Other: ≥ 1 serving/day		Daily
**Whole grains**	1–2 servings/meal	7–8 servings/day	≥ 3 servings/day	Bread: 4–6 slices/day Cereal: 1.5 servings/day Pasta: 3 servings/week β-glucan-rich foods: 3 g/d	Daily
**Dairy**	Low-fat: 2 servings/day	Low- or non-fat: 2–3 servings/day	Cheese: < 1 serving/week Butter: < 1 Tbsp/day	Low-fat milk: ≤ 5 dL/day Cheese: for cooking ^b^	Yogurt: daily to weekly
**Nuts, seeds, and legumes**	Olives/nuts/seeds: 1–2 servings/day Legumes: ≥ 2 servings/week	4–5 servings/week	Nuts: ≥ 5 servings/week Beans: > 3 servings/week	Nuts (mostly almonds): 15 g/day	Daily
**Beef, pork, ham, lamb, veal, poultry**	Red meat: < 2 servings/week Processed meat: ≤ 1 servings/week White meat: 2 servings/week	Lean protein: ≤ 2 servings/day	Red meat: < 4 servings/week	Meat: ≤ 500 g/week	Red meat: infrequent
			Poultry: ≥ 2 servings/week	Poultry: ≤ 300 g/week	Poultry: Daily to weekly
**Fish/seafood**	≥ 2 servings/week		≥ 1 serving/week	3–5 servings/week	2 servings/week
**Fats, oils, and salad dressing**	Olive oil: 1–2 servings/meal	2–3 servings /day	Olive oil as primary oil	5 g/bread slice 0.5 dL/day as dressing	Healthy cooking oils: daily to weekly
**Sweets**	≤ 2 servings/week	≤ 5 servings/week	Pastries & sweets: < 5 servings/week	On weekends	Infrequent
**Other**	Eggs: 2–4 servings/week Potatoes: ≤ 3 servings//week	Sodium < 2,300 mg/day	Fried or fast food: < 1 serving/week	Eggs: Stay within daily recommended cholesterol intake Fruit/vegetable juice: 4 dL/week	Eggs: daily to weekly
**Alcohol**	Wine: in moderation	Women: ≤ 1 drink/day Men: ≤ 2 drinks/day	1 glass/day	Habitual amount	In moderation

^a^ Recommendations shown here are based on a 2000 calorie per day eating plan. ^b^ Contribution of total fat and quality of fat from cheese to stay within the recommended daily intake.
